# Supplementary figures and images for: Gut microbiota diversity repeatedly diminishes over time following maintenance infliximab infusions in paediatric IBD patients
Source: PLoS One. 2024 Dec 12;19(12):e0311604. doi: 10.1371/journal.pone.0311604 (PMC11637414; doi:10.1371/journal.pone.0311604)

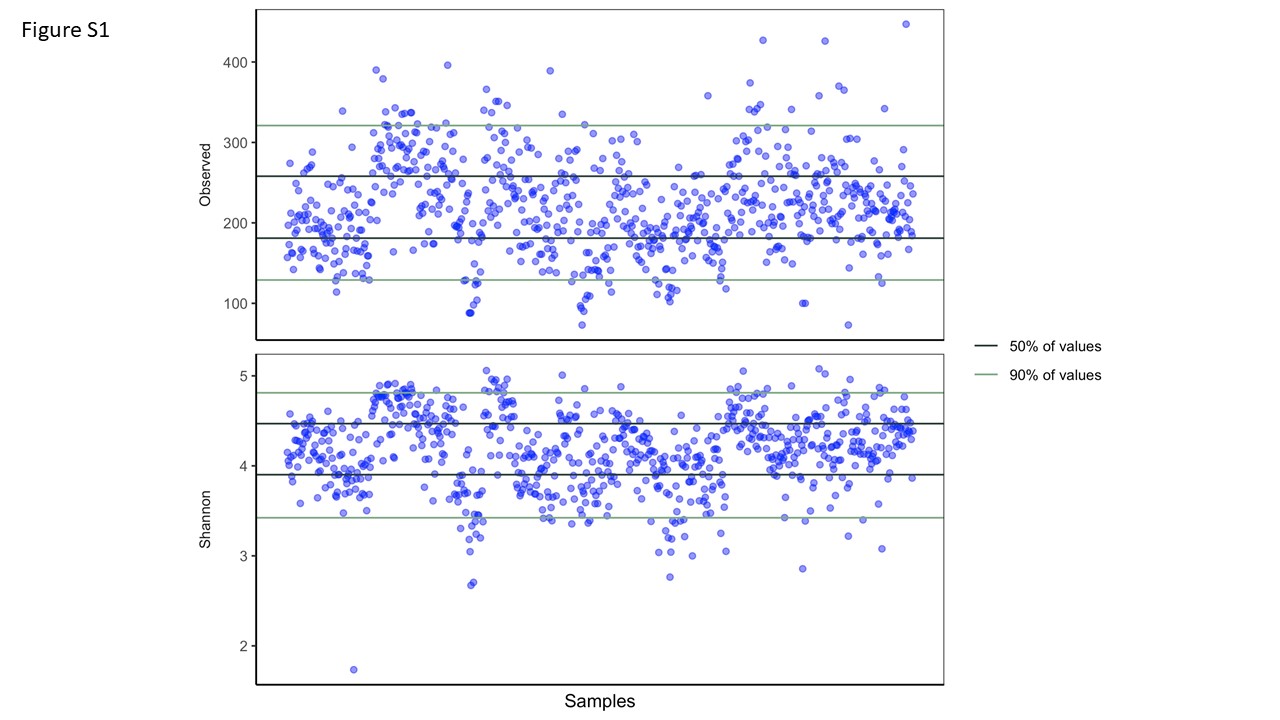

Supplement: S1 Fig — (JPG) [file pone.0311604.s004.jpg]

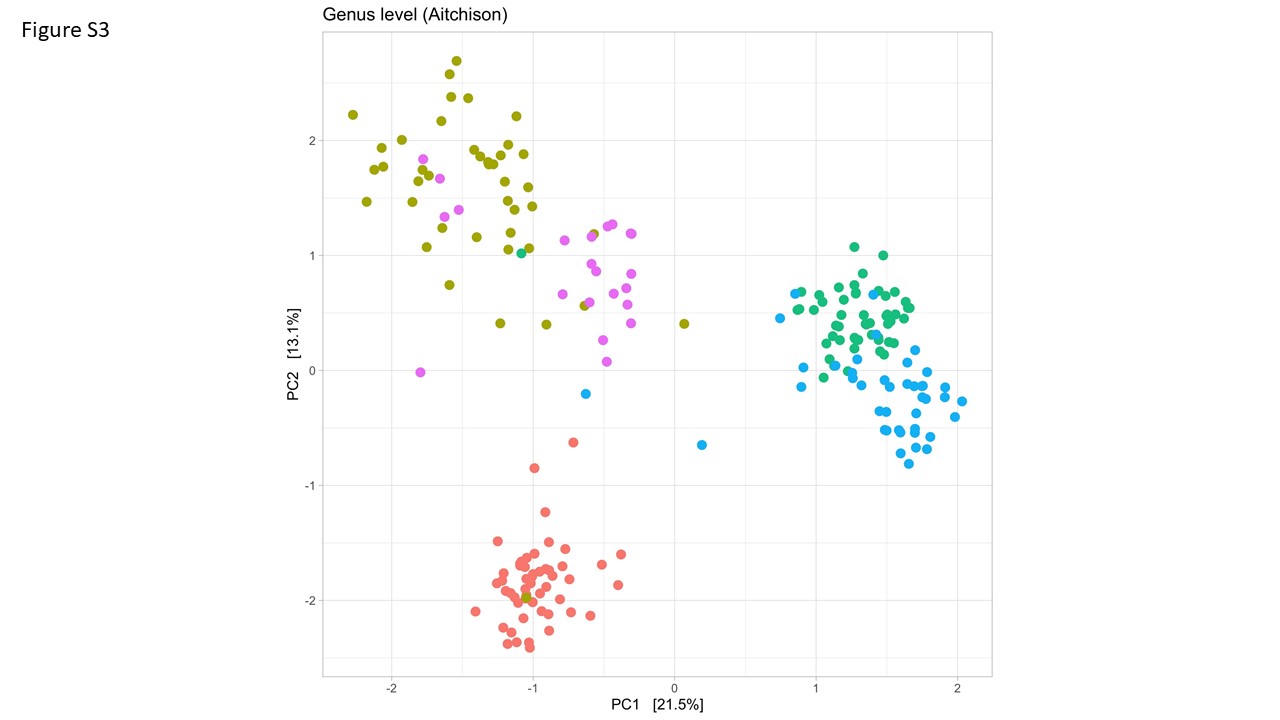

Supplement: S2 Fig — (JPG) [file pone.0311604.s005.jpg]

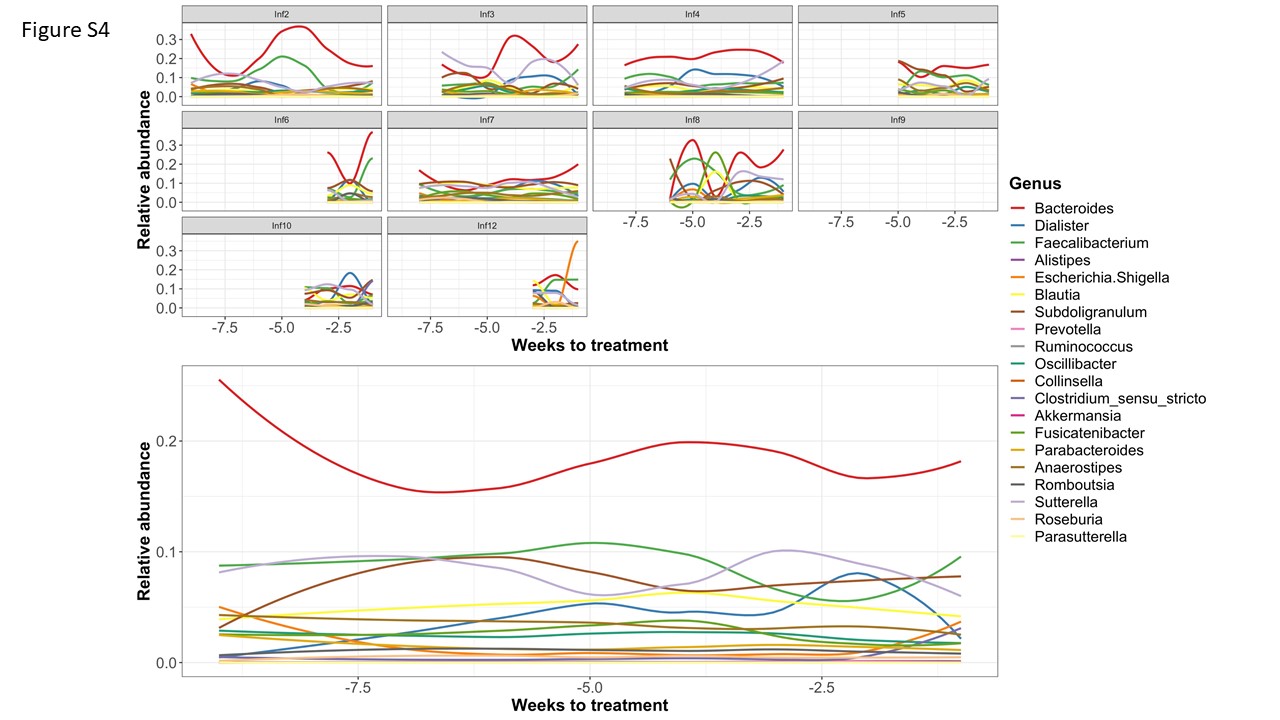

Supplement: S3 Fig — (JPG) [file pone.0311604.s006.jpg]

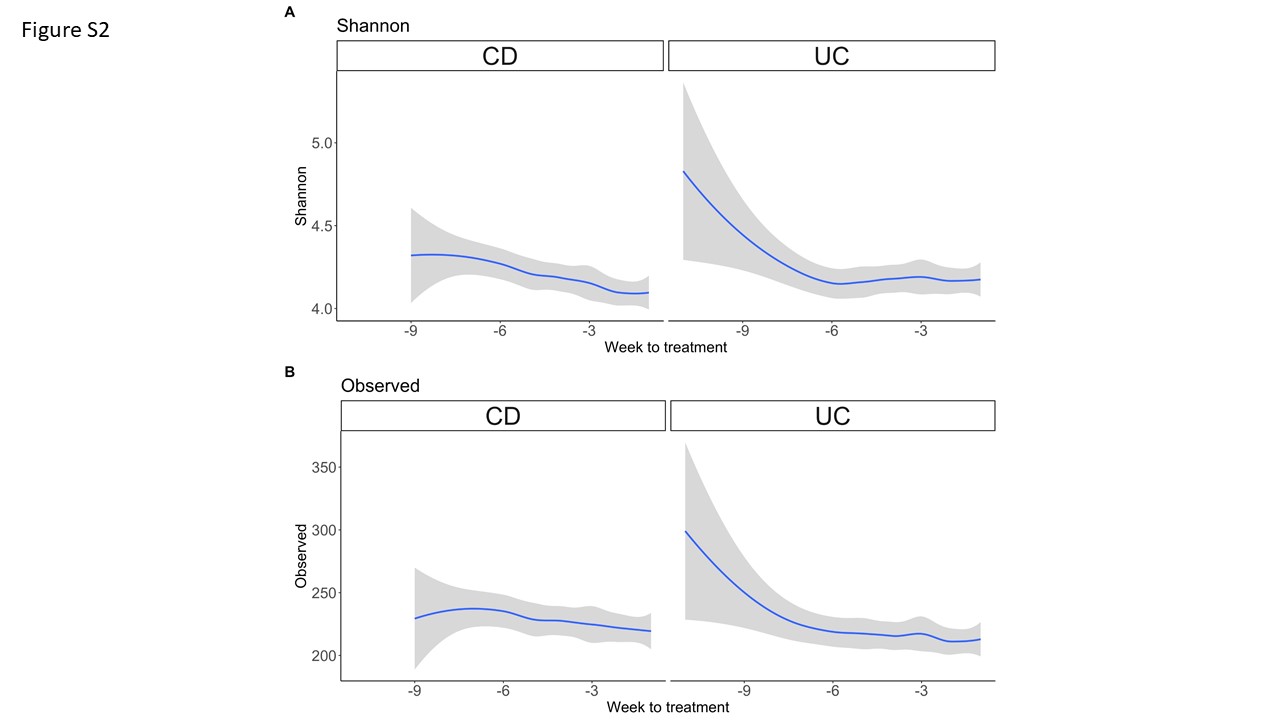

Supplement: S4 Fig — (JPG) [file pone.0311604.s007.jpg]

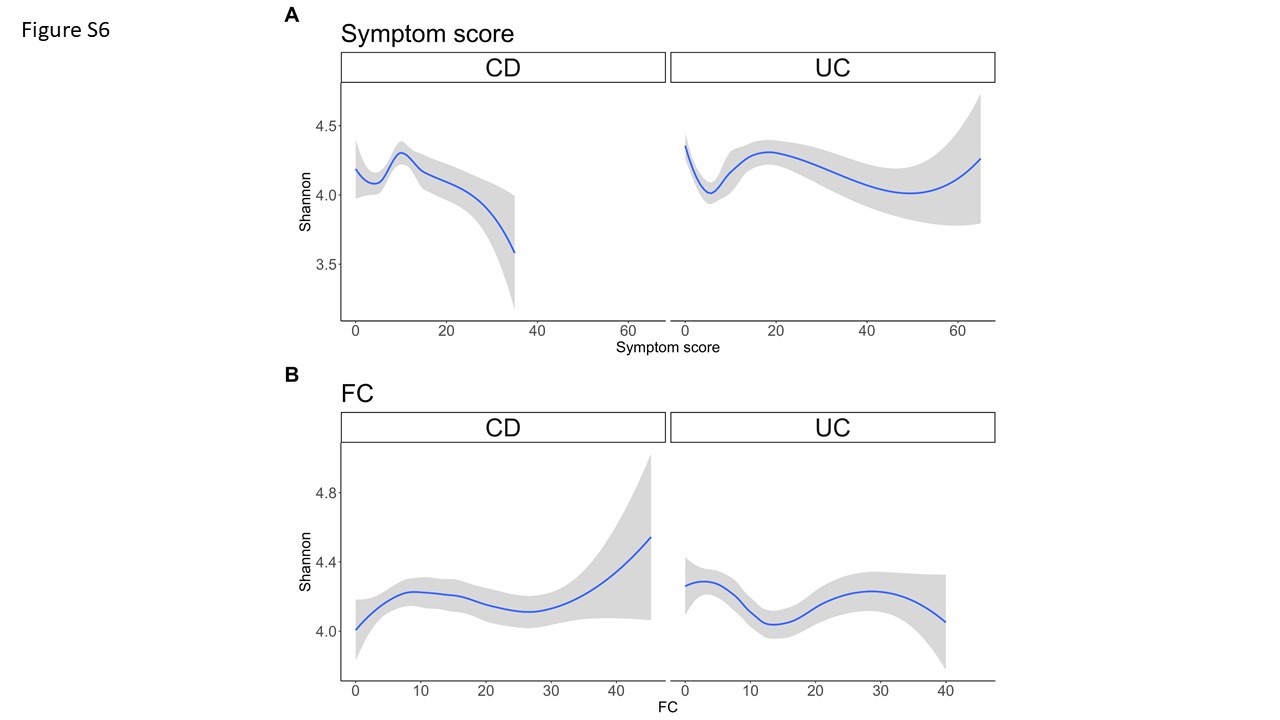

Supplement: S5 Fig — Development of Shannon diversity across symptom scores (A) and FC values (B). (JPG) [file pone.0311604.s008.jpg]

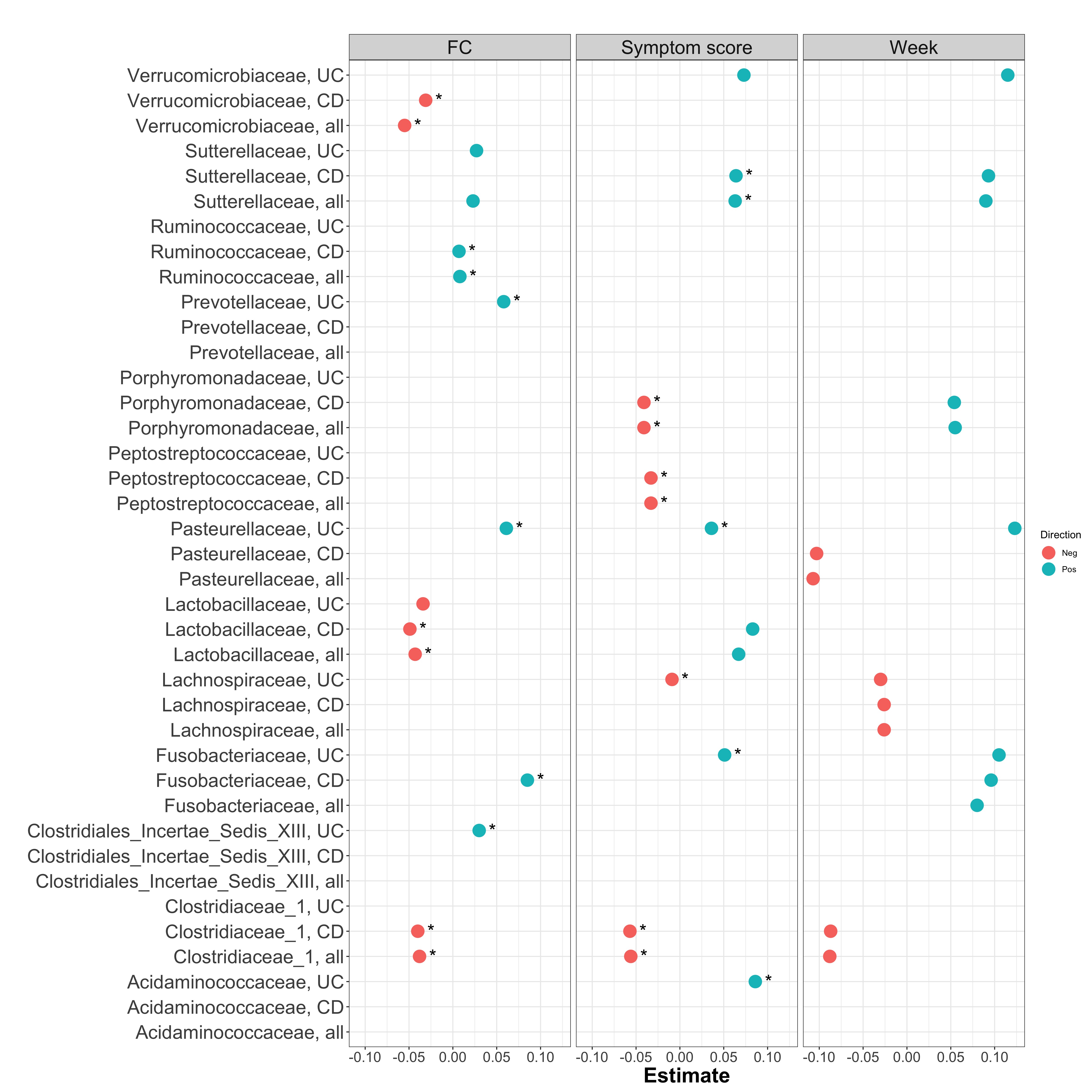

Supplement: S6 Fig — (JPEG) [file pone.0311604.s009.jpeg]
