# Supplementary material for: Gut microbiota diversity repeatedly diminishes over time following maintenance infliximab infusions in paediatric IBD patients
Source: PLoS One. 2024 Dec 12;19(12):e0311604. doi: 10.1371/journal.pone.0311604 (PMC11637414; doi:10.1371/journal.pone.0311604)
Supplement: Attachment — Submitted filename: plos_one_review_20240218.pdf [file pone.0311604.s010.pdf]

Review of the article submitted Katrine Carlsen, MD, PhD entitled Gut microbiota diversity repeatedly diminishes over time following maintenance infliximab infusions in paediatric IBD patients

Made by: Jakub Hurych, M.D.

Overall summary: The study itself is interesting because of its longitudinal design and a decent amount of samples for the analysis. However, it is clearly visible that the study was originally done many years ago, and therefore, many caveats in regard to the microbiome analysis occurred. Mostly importantly, it's the time from sampling to deep freezing of stool samples. Sending samples by mail is ok for calprotectin analysis, but it's definitely worthless for microbiome testing if not sent on dry ice or at least in the cooling container, which was not specified. Squeezing another paper from an otherwise overwhelming sample collection and a great study might, therefore, not be impossible.

Also, there are a few minor issues in terms of the terminology of microbiome analysis and interpreting the p-values over 0,05 as significant, as seen in the table. Also, the literature review seems insufficient, as a few recent papers from respected journals are missing in the Introduction and Discussion.

In summary, I would strongly argue for clarifying my above-mentioned concerns and adjusting the paper in terms of the issues mentioned below. As the row numbering was surprisingly not present, I only referred to the page of the PDF I received.

The reviewer's comments are mentioned in the table below.

Page in PDF	Comment
3	Methods: Distribution of IBD should be mentioned already here.
3	Age: Median range missing
3	Observed: It's usually called Observed species
3	Firmicutes Anaerostipes: misleading, I would suggest to put in brackets after the genus level, simmilarily with Proteobacteria below and again on page 16
5	Symptoms include (s missing)
5	? extraintestinal symptoms and name them in the brackets?
5	“less gut diversity” It‘s ussualy called lower gut microbiome diversity
5	Better: "its treatment"?
5	Last sentence of Introduction: Study by Hurych et al published in 2023 used follow up to 3 months, it should be mentioned. Also, they used six samples per patient, which might not be considered as few. As they report, by this design the fluctuation of the microbiome beta diversity was described in a relative good way.<br><br>Also, there is a study by Ventin-Holmberg et al from JCC, wherte they also described some changes in longitudinal design.<br><br>Neither of those studies are cited. Therefore, I would highly suggest the author to rethink the whole last sentence, its
6	12 – usually written in words
6	Was the sample mailed by post on dry ice or at least in a cooling container? If not, then is worthless for microbiome analysis. Please, specify.
6	Degrees Celsius: why not to use the abbreviation?
7	R version: The latest version is 4.3.3. Why such an old one?
7	“ As the age span was limited and gender was balanced, these variables were not included in the statistical analyses.“ – where is the proof?
8	There are more alpha diversity indices. Why only Shannon and Observed species were used?
11	The microbiome's composition was analysed – it must be specifed
11	Again, why only Shannon? It puts more weight on richness than on evenness unlike the other indices, tehredore I strongly suggest the author to include theother alpha diversity metrics as well (at least Chao1 and Simpson)
12	„negative trend“ – replace by: similar, but not significant negative trend
12	Using the PCA analysis shoud be mentioned in the methods.
13	I would suggest to use a different term than „Results for weeks since treatment“. Time since treatment? Also the name of the paragraph is not in conlusion with previous on. I suggest Analysis of the .
13	Prior studies …. - It sounds more like a discussion.
13	Significantly different association - Were the findings corrected for number of tested taxa? Ther is p. adj but it is not described for what.<br><br>If not, they should be or at least it should be mentione dwhy this did not happen.
13	Correct: Trending but non-sugnificant difference
13	p.adj below 0.1 - This is not significant.

13	p.adj – adjusted for what
13	Association in CD and across all patients (*p*<0.1) - What kind of association is this, please?
14	(CD, *p*=0.057) – this is no association
14	Further genera showed a nominal association with weeks since treatment (glmm, *p*<0.1) – no association
14	namely *Akkermansia* (positive association in UC), *Blautia* and *Clostridium* sensu stricto (negative association in CD and all patients in total), *Prevotella* (positive association in CD and all patients), *Subdoligranulum* (positive association in CD) – where are the p-values to confirm the statement?
15	Discussion: There are only two studies in PIBD mentioned, but there are more, see comment in the ntroduction. This first two paragraphs should be corrected.
16	Faecalibacterium – in italics, Firmicutes has a typo
16	Again, only few studies are cited, but there are more. The recent study by Hurych et al is also refering to the alpha diversity: No significant association of the alpha diversity indices was noted with the mucosal healing (assessed by MINI index three months after commencing anti-TNF) among the CD patients on anti-TNF therapy.<br><br>As the MINI is mostly influenced by FCPT, it should be mentioned.
16	Rename to: Single taxa analysis
16	Include more studies in the single taxa analysus discussion
17	Limitations: sending samples by post - this is a huge limitation. As mentioned aboved, this must be explained.
18	Our results support… - This is a strong statement considering the results. Only very few were real association, and if so, they were very cloes to the 0,05 mark
