# Supplementary material for: Gut microbiota diversity repeatedly diminishes over time following maintenance infliximab infusions in paediatric IBD patients
Source: PLoS One. 2024 Dec 12;19(12):e0311604. doi: 10.1371/journal.pone.0311604 (PMC11637414; doi:10.1371/journal.pone.0311604)
Supplement: Attachment — Submitted filename: plos_one_review_20240731.pdf [file pone.0311604.s012.pdf]

Second review of the article submitted by Katrine Carlsen, MD, PhD entitled "Gut microbiota diversity repeatedly diminishes over time following maintenance infliximab infusions in paediatric IBD patients"

Made by: Jakub Hurych, M.D., Ph.D.

Sent: July 31st, 2024

Overall summary: Firstly, I would like to express my appreciation for the authors' thoughtful responses to my comments and suggestions. The revised paper is notably improved and more accessible compared to the original version. I have only one concern regarding the interpretation of the relationship between the taxa *Parasutterella*, *Fusicatenibacter*, and *Anaerostipes* with the duration since treatment in the CD group. Specifically, the distinction between statistical significance and nominal significance should be carefully maintained in both the abstract and discussion sections, as detailed below. The mention of an association with a p-value of less than 0.1, rather than the conventional threshold of less than 0.05, appears unconventional.

Secondly, I would like to sincerely apologize to the authors if the tone of some of my comments in the initial review seemed harsh; that was not my intention. I appreciate the thorough and constructive responses the authors provided despite this.

Although the article by Carlsen et al. has some limitations, these are thoroughly discussed within the text. Overall, the study adds valuable insight into the topic of the pIBD microbiome, particularly concerning the diversity dynamics related to infliximab treatment.

The reviewer's specific comments are summarized in the table below.

Row	Comment
79 (and also 392-409 and 480-495)	1."(p.adj<0.1) " is no statistical association, this is only below 0.05.<br><br>There is no doubt about an association between the genera and the time since the last infusion in UC (p.adj=0.037, 0.037, and 0.037; line 391). But for CD, there is no mention about statistical significance between the three genera and time since treatment, as line 395 shows a p-value <0.1 – it is only nominal and not statistical; I would mention it in the results, but discuss and highlight only the statistically significant results.<br><br>Similarilly, in the discussion, all is discussed as a clear significance between the three genera and time since treatment in both UC and CD. But again, this is not supported by the results. I would like to see Table 1 with all the modelling results, but I was not given the oportunity to see it (I asked via email, but with no response), unfortunately. But if the adjusted p-value for the relation between *Parasuterella*, *Fusicatenibacter* and *Anaerostipes* in CD is not below 0.05, it is not statistically significant and should be discussed as that.<br><br>To sum up, I would kindly ask the authors for either an explanation to or a correction in this regard.
97	It seems confusing: … "before diversity decreases again" - I got the point, but it took me a while; I would suggest the authors consider different wording to make the diversity dynamics statement clear enough.
425-6	I appreciate the idea of summarising the main point of the article, but it seems a word might be missing or I did not understood the meaning.
