# Supplementary material for: Gut microbiota diversity repeatedly diminishes over time following maintenance infliximab infusions in paediatric IBD patients
Source: PLoS One. 2024 Dec 12;19(12):e0311604. doi: 10.1371/journal.pone.0311604 (PMC11637414; doi:10.1371/journal.pone.0311604)
Supplement: Attachment — Submitted filename: Reply to reviewers 28.6.24.docx [file pone.0311604.s011.docx]

**Review Comments to the Author**

Please use the space provided to explain your answers to the questions above. You may also include additional comments for the author, including concerns about dual publication, research ethics, or publication ethics. (Please upload your review as an attachment if it exceeds 20,000 characters)

**Reviewer #1:** The study itself is interesting because of its longitudinal design and a decent amount of samples for the analysis. However, it is clearly visible that the study was originally done many years ago, and therefore, many caveats in regard to the microbiome analysis occurred. Mostly importantly, it’s the time from sampling to deep freezing of stool samples. Sending samples by mail is ok for calprotectin analysis, but it’s definitely worthless for microbiome testing if not sent on dry ice or at least in the cooling container, which was not specified. Squeezing another paper from an otherwise overwhelming sample collection and a great study might, therefore, not be impossible.

**Reply to reviewer comment:**

We thank the reviewer very much for the observations and can confirm the manuscript has been long underway not least due to the +9 months in review at PLOSONE.

The samples were indeed shipped at room temperature and thus been at RT typically for 1-3 days. Had the project been planned today we would indeed have selected to use a stabilizer as good options have since been made available and tested in depth. However, while we did discuss the point on engaging into this project, we decided that the unique nature of the samples with matched metadata could provide key information, and that the size and longitudinal nature of the cohort could counterbalance the issues arising from variance introduced in shipment. We rely on publications on the matter that show that storage at RT for shorter timeframes introduce minimal variance, and while shipment at RT with no additive often perform worse than those immediately frozen, they do not perform badly (Lauber 2010; Cardona 2012). Thus, while the approach is indeed less optimal to other approach, the intraclass correlation coefficient (ICC) for alpha diversity measures and most single taxa (Fig 1 in Byrd 2019) were high, also for these samples. We agree that is an important point to consider when reading the paper and must be clear from the discussion. We have made edits accordingly in the manuscript.

**The above mentioned references:**

Lauber, Christian L et al. “Effect of storage conditions on the assessment of bacterial community structure in soil and human-associated samples.” *FEMS microbiology letters* vol. 307,1 (2010): 80-6. doi:10.1111/j.1574-6968.2010.01965.x

Cardona, S., Eck, A., Cassellas, M. *et al.* Storage conditions of intestinal microbiota matter in metagenomic analysis. *BMC Microbiol* **12**, 158 (2012). <https://doi.org/10.1186/1471-2180-12-158>

Byrd, Doratha A et al. “Reproducibility, stability, and accuracy of microbial profiles by fecal sample collection method in three distinct populations.” *PloS one* vol. 14,11 e0224757. 18 Nov. 2019, doi:10.1371/journal.pone.0224757

Also, there are a few minor issues in terms of the terminology of microbiome analysis and interpreting the p-values over 0.05 as significant, as seen in the table.

**Reply to reviewer comment:**

Please see our replies to these issues below.

Also, the literature review seems insufficient, as a few recent papers from respected journals are missing in the Introduction and Discussion.

**Reply to reviewer comment:**

We agree and this has been done. Please, if we missed any work deemed essential due not hesitate to mention which. Thanks for noticing.

In summary, I would strongly argue for clarifying my above-mentioned concerns and adjusting the paper in terms of the issues mentioned below. As the row numbering was surprisingly not present, I only referred to the page of the PDF I received. The reviewer’s comments are mentioned in detail in the attached PDF.

**Replies to comments from PDF from Reviewer #1**

3 Methods: Distribution of IBD should be mentioned already here.

**Authors reply:**

We have now added the distribution of Crohn´s Disease and ulcerative colitis in the abstract.

3 Age: Median range missing

**Authors reply:**

Information have been added.

3 Observed: It's usually called Observed species

**Authors reply:**

This has been corrected.

3 Firmicutes Anaerostipes: misleading, I would suggest to put in brackets afterthe genus level, simmilarily with Proteobacteria below and again on page 16

**Authors reply:**

We agree this was not an ideal approach and has corrected as suggested.

5 Symptoms include (s missing)

**Authors reply:**

This has been corrected.

5 ? extraintestinal symptoms and name them in the brackets?

**Authors reply:**

Please find added information in the text.

5 “less gut diversity” It‘s ussualy called lower gut microbiome diversity

**Authors reply:**

The text has been changed.

5 Better: "its treatment"?

**Authors reply:**

The text has been changed.

5 Last sentence of Introduction: Study by Hurych et al published in 2023 used follow up to 3 months, it should be mentioned. Also, they used six samples per patient, which might not be considered as few. As they report, by this design the fluctuation of the microbiome beta diversity was described in a relative

good way.Also, there is a study by Ventin-Holmberg et al from JCC, where they also described some changes in longitudinal design. Neither of those studies are cited. Therefore, I would highly suggest the author to rethink the whole last sentence, its

**Authors reply:**

Thanks for noticing. During the submission-process (9+ months) new relevant references have emerged. Please see the updated introduction and discussion section, as well as the reference list.

6 12 – usually written in words

**Authors reply:**

We have changed many occurrences of the number 12 to now be written in letters to adhere to the reviewers request even as we consider the rule to apply only to number up to 10.

6 Was the sample mailed by post on dry ice or at least in a cooling container? If not, then is worthless for microbiome analysis. Please, specify.

**Authors reply:**

Please see out points on this subject in beginning of “5. Review Comments to the Author”

6 Degrees Celsius: why not to use the abbreviation?

**Authors reply:**

We changed to using the abbreviation, not giving an explanation for this abbreviation, however, as it was used only once.

7 R version: The latest version is 4.3.3. Why such an old one?

**Authors reply:**

This also reflect that the project has been ongoing for quite some time.

7 “ As the age span was limited and gender was balanced, these variables were not included in the statistical analyses.“ – where is the proof?

**Authors reply:**

The supporting values are found in Table 1. We have added reference to the table to the sentence.

8 There are more alpha diversity indices. Why only Shannon and Observed species were used?

**Authors reply:**

There are indeed many alpha diversity indices, and one needs to select a subset to use. While each measure is developed to capture different aspects of the diversity, many of the commonly used measures are highly correlated as is shown in figure 1 of (Hagerty 2020). Shannon is a commonly used diversity measure that correlate well with other common measures such as Chao1, ace, faiths pd and Simpson. Shannon encompasses information on both richness and evenness while observed richness is a simple measure of the number of different species detected. Thus, we find that using these two measures reflect much of the alpha diversity and that including more measures would complicate the reading of the manuscript as many summary stats would have to be reported for each analysis of alpha diversity.

We have previously reported on more measures and in the review process been accused of performing a fishing expedition. Thus, we find performing the analyses with the two measures is the optimal sweet spot, and we hope that we can convince the reviewer of this proposition.

*Hagerty SL, Hutchison KE, Lowry CA, Bryan AD (2020) An empirically derived method for measuring human gut microbiome alpha diversity: Demonstrated utility in predicting health-related outcomes among a human clinical sample. PLoSONE 15(3): e0229204*

11 The microbiome's composition was analyzed – it must be specified

**Authors reply:**

Indeed an analysis of overall composition was performed as detailed in methods and with the main results described in the section on p12 that reads “Analysis of the overall community composition (beta-diversity) and weeks-to-treatment identified a significant association (permanova analysis using vegan::adonis function with Aitchison distances, including 671 samples and controlling for diagnosis (CD or UC) and 5-ASA; R2=0.004, p=0.047). Correspondingly, both symptom score and FC were associated with overall community composition (symptom score R2=0.011, p=0.018; FC R2=0.018, p=0.001). As such, analysis of the overall community composition supported an association between the microbiome composition and the outcome measures, with the alpha diversity analyses showing that the gut microbial diversity becomes less diverse as the time elapsed since the last treatment increases. Visual inspection of the overall community composition (using principal components ordination) showed a general clustering of samples from the same subject, as expected, based on prior analysis of human gut microbiomes across individuals (see Figure S3 which show clustering by subject for a selected subset of five patients).”

11 Again, why only Shannon? It puts more weight on richness than on evenness unlike the other indices, therefore I strongly suggest the author to include the other alpha diversity metrics as well (at least Chao1 and Simpson)

**Authors reply:**

Please see our response to point 8 above.

12 „negative trend“ – replace by: similar, but not significant negative trend

**Authors reply:**

Thank you for the good suggestion, which has been inserted.

12 Using the PCA analysis should be mentioned in the methods.

**Authors reply:**

That was added now to the method part that describes the other beta-diversity-based analyses.

13 I would suggest to use a different term than „Results for weeks since treatment“. Time since treatment? Also the name of the paragraph is not in conlusion with previous on. I suggest Analysis of the .

**Authors reply:**

Thank you for the suggestion, the text has been changed.

13 Prior studies ….

- It sounds more like a discussion.

**Authors reply:**

We have mentioned “Prior studies…” to give the reader the reason for why we initiate this section with analysis aimed at understanding the possible role of this difference for our analyses, before diving into the analyses that are the aim of this study.

13 Significantly different association - Were the findings corrected for number of tested taxa? Ther is p. adj but it is not described for what. If not, they should be or at least it should be mentione dwhy this did not happen.

**Authors reply:**

When testing across multiple taxa adjustment was performed using stats::p.adjust in R and the adjusted p-values given with p.adj. This information has now been added to methods.

13 Correct: Trending but non-sugnificant difference

**Authors reply:**

We have specified the trending statement with “non-significant” and stick to “nominally significant” when discussing results with nominal p<0.05.

13 p.adj below 0.1 - This is not significant.13 p.adj – adjusted for what

**Authors reply:** We have worked through the manuscript updating the wording to comply with the above-mentioned logic. The p.adj is multiple testing corrected p-values (adjusted p-values) as now specified in methods.

13 Association in CD and across all patients (p<0.1) - What kind of association is

this, please?

14 (CD, p=0.057) – this is no association

14 Further genera showed a nominal association with weeks since treatment

(glmm, p<0.1) – no association

**Authors reply:**

For points 13-14 we have worked through the results making sure the manuscript conforms with the general notion of significance being p or p.adj <0.05.

14 namely Akkermansia (positive association in UC), Blautia and Clostridium sensu stricto (negative association in CD and all patients in total), Prevotella (positive association in CD and all patients), Subdoligranulum (positive association in CD) – where are the p-values

to confirm the statement?

**Authors reply:** Reference to the table S1 that holds all summary stats has been added.

15 Discussion: There are only two studies in PIBD mentioned, but there are more, see comment in the introduction. This first two paragraphs should be corrected.

**Authors reply:**

The introduction and discussion have been updated.

16 Faecalibacterium – in italics, Firmicutes has a typo

**Authors reply:**

Has been corrected

16 Again, only few studies are cited, but there are more. The recent study by Hurych et al is also refering to the alpha diversity: No significant association of the alpha diversity indices was noted with the mucosal healing (assessed by MINI index three months after commencing anti-TNF) among the CD patients on anti-TNF therapy. As the MINI is mostly influenced by FCPT, it should be mentioned.

**Authors reply:**

Please find the introduction and discussion updated with more studies referenced. While more studies have analyzed the microbiome in relation to IBD and/or anti-TNF, few has the longitudinal aspect or analyzed children. We find that there are only few studies with which we can compare results, and hope the reviewer find the updates we made are sufficient. Specifically for the last part of the comment, we are unsure what is meant.

16 Rename to: Single taxa analysis

**Authors reply:**

This has been added.

16 Include more studies in the single taxa analyses discussion

**Authors reply:**

With respect to the length of the discussion, references have been added.

17 Limitations: sending samples by post - this is a huge limitation. As mentioned above, this must be explained.

**Authors reply:**

Additional information has been added to this limitation section.

18 Our results support…

- This is a strong statement considering the results. Only very few were real association, and if so, they were very close to the 0,05 mark

**Authors reply:**

The sentence has been changed.

**Reviewer #2:** The manuscript presents a longitudinal study investigating the changes in the alpha diversity of gut microbiome in pediatric inflammatory bowel disease (IBD) patients in relation to infliximab (IFX)
treatment, fecal calprotectin (FC) levels, and symptom scores. The longitudinal approach of the study is
a key strength, as it provides insights into the dynamic nature of the gut microbiome in the context of
IBD management. Further, the authors employed appropriate statistical methods, including mixed linear
models, PERMANOVA, and negative binomial generalized linear mixed models, to analyze the
microbiome data and account for potential confounding factors. This rigorous analytical approach
strengthens the reliability of the findings. This reviewer suggests addressing a few concerns to make the manuscript strong.

**Reply to reviewer comment:**

We thank the reviewer very much for recognizing the strengths of the study and unique possibilities to investigate aspects of microbiome and treatment interactions originating from this dataset. We have done our best to address the points made below and thank the reviewer for having such keen eyes for the details.

**Major concern:**

The authors have mostly focused on alpha diversity assessment in these samples, which
provides a limited understanding of the microbiome changes. It would be beneficial to include
more information on beta diversity to capture the overall community composition changes in
relation to IFX treatment, FC levels, and symptom scores.

**Authors reply:**

Indeed an analyses of the association between the outcome measures and overall composition is relevant and was performed as detailed in methods and with the main results described in the section on p12 that reads “Analysis of the overall community composition (beta-diversity) and weeks-to-treatment identified a significant association (permanova analysis using vegan::adonis function with Aitchison distances, including 671 samples and controlling for diagnosis (CD or UC) and 5-ASA; R2=0.004, p=0.047). Correspondingly, both symptom score and FC were associated with overall community composition (symptom score R2=0.011, p=0.018; FC R2=0.018, p=0.001). As such, analysis of the overall community composition supported an association between the microbiome composition and the outcome measures, with the alpha diversity analyses showing that the gut microbial diversity becomes less diverse as the time elapsed since the last treatment increases. Visual inspection of the overall community composition (using principal components ordination) showed a general clustering of samples from the same subject, as expected, based on prior analysis of human gut microbiomes across individuals (see Figure S3 which show clustering by subject for a selected subset of five patients).”

The authors should provide a justification for the observed pattern of alpha diversity increasing
in the first 4 weeks after IFX treatment but decreasing thereafter. A more in-depth discussion of
the potential mechanisms and implications would strengthen the manuscript.

**Authors reply:**

We interpret the observation that alpha-diversity first increases and then, as time since treatment increases, as a reflection of a fast response by the microbiome, either directly to the treatment or indirectly to an improvement of the gut, that then diminishes. Please find elaboration in the discussion.

The authors should clearly state the IFX dosing protocol used in the study, as this information is
crucial for interpreting the results.

**Authors reply:**

Please find added information regarding dosing in section “Study subjects, sample collection and data overview”.

Additionally, the authors should discuss the potential impact of the variable treatment intervals on the microbiome analysis. It would be helpful to know if the microbiome changes observed were consistent across the different treatment intervals or if there were any differences based on the duration between IFX infusions.

**Authors reply:**

Patients who needed treatment before week 12 due to increased disease activity had lower alpha diversity, which also appeared to decrease further as the time elapsed since the last treatment, compared to patients in remission during the 12-week IFX interval. However, statistical analysis (mixed model analysis, adjusting for patient dependence, with random term for patient, *p*>0.1) did not detect a significant difference in the relationship between alpha diversity and weeks since treatment between the two groups. This is illustrated in figure 2.

We hope this answer covers your question (result section and discussion).

The authors mentions that treatment intervals with IFX were determined by the Total
Inflammatory Burden Score (TIBS), which was based on fecal calprotectin (FC) levels and
symptom scores, and the intervals ranged from a minimum of 4 weeks to a maximum of 12
weeks. However, it's not clear how many patients out of 25 received the IFX treatment at what
week interval; the 4 – 12 weeks' timeframe is broad. It will be helpful to include this
information and if some patients received multiple IFX infusions in the study, then it should be
described appropriately.

**Authors reply:**

This is indeed an important information to have when reading the manuscript and we have now added information regarding the intervals and number of treatments per patient in the results section “Project overview”.

The authors should explain how the Total Inflammatory Burden Score (TIBS) (FC/symptom
score) was calculated. It looks like for the symptom scores, two different indices were used for
UC and CD patients, and additional information may help in understanding the scoring system.

**Authors reply:**

Please find added text for clarification of the algorithm in Materials and Methods section, “Study subjects, sample collection and data overview”.

The authors have accounted for the confounding effects of treatment with 5-aminosalicylic acid
(5-ASA) and thiopurines. However, the study did not account for other factors that could
influence the gut microbiome, such as diet and lifestyle factors. The effect of such factors on
the microbiome should be acknowledged in the discussion.

**Authors reply:**

Diet and lifestyle are relevant, albeit challenging, factors to consider in microbiome studies. Unfortunately, we did not have data representing those factors for this cohort. We do however not find this to be a major concern for this study as lifestyle and diet is unlikely to have changed dramatically during these short cyclic periods. We have added text on these matters under the section on limitations.

**Minor concerns:**

The abstract should provide a more comprehensive background and context for the study, rather than
simply stating the aim. A brief introduction to the relevance of the gut microbiome in inflammatory
bowel disease (IBD) and the importance of understanding its dynamics in relation to treatment would
help readers better appreciate the significance of the research question being addressed.

**Authors reply:**

We agree that such introduction can help the reader see the value of the study, albeit it is difficult to properly give such background and context in a few sentences. We have added a bit of text and hope it meets the intention.

Please provide the primer sequences used to amplify V1V2 region of 16S rRNA gene in this study.

**Authors reply:**

**The primers used were “**27F-AGAGTTTGATCCTGGCTCAG, 338R-TGCTGCCTCCCGTAGGAG” and this information has been added to the manuscript.

Please explain which 16S database was used for taxonomic assignments.
PCoA plot with just five representative samples is Okay (figure S3), but specifically mention this in the
manuscript.

**Authors reply:**

We have added the information that the figure includes only 5 patients when referring to the supplementary figure.

The authors should consistently use appropriate "genus-level" or "genera level" taxonomic
identifiers throughout the manuscript.

**Authors reply:**

We apologies for not consistently using appropriate terminology. We have made edits were deemed appropriate and hope we did detect all issues.

In the discussion section "FC levels and alpha diversity measure" the name of the species and the phylum is misspelled, it should be Faecalibacterium prausnitzii (Firmicute).

**Authors reply:**

The error has been corrected; sorry for the inconvenience.

Figures:

Figure 1 is not referred to in the result section. The authors should either refer to this figure or
consider removing it if it is not essential.

**Authors reply:**

We have added a reference to the figure to the text in the section “Changes in microbiome diversity in the lead-up to treatment”.

For Figure 1, the authors should explain why phyla with over 2% relative abundance were
considered.

**Authors reply:**

An explanation of the filtering has now been added to the figure legend.

Figures 2A and 2B are not referred to in the result section. The authors should either refer to
these figures or consider removing them if they are not essential.

**Authors reply:**

We Apologize for this mistake and have added a reference to Figure 2.

Figure 4 is not referred to in the result section. Also, the Y-axis title should be 'Taxa relative
abundance' (there is a typo).

**Authors reply:**

We have now added reference to the figure and corrected the typo.

Please provide the legends for figure S3.

**Authors reply:**

A legend to explain the color of the dots would not be informative as there is no information given by stating subjects IDs. We elaborated in the figure legend that each individual of the 5 subjects is given a unique color.

For Figures 3 and S5, please add the legends within the figures.

**Authors reply:**

The color legend has now been added.

Supplementary tables 1, 2, and 3 could not be found. The authors should provide all supplementary materials and properly referenced them in the main text.

**Authors reply:**

There must have been a technical error. We apologies for the inconvenience. We have uploaded them again and checked they are referred to in the text.
