# Supplementary material for: Gut microbiota diversity repeatedly diminishes over time following maintenance infliximab infusions in paediatric IBD patients
Source: PLoS One. 2024 Dec 12;19(12):e0311604. doi: 10.1371/journal.pone.0311604 (PMC11637414; doi:10.1371/journal.pone.0311604)
Supplement: Attachment — Submitted filename: Reply to reviewers 05.08.2024.docx [file pone.0311604.s013.docx]

**Review Comments to the Author**

 There was only a single comment by Reviewer #1 about the reporting of significant taxa associated with time elapsed since last infusion. I believe that the indicated cutoff of p.adj<0.1 is fine, but the final sentence of the Results subsection of the Abstract is unclear. It suggests that all three taxa achieved this significance threshold in the full cohort, but based on the Results and Table S1 only Parasutterella was significant across the full cohort (padj=10^-10), while all three taxa were significant specifically in UC (at a threshold of p.adj<0.05) but not CD in subset analyses. Please edit this sentence and possibly the Results section (under “Analysis of time (weeks) since treatment for single taxa“) to clarify the findings of the analysis on taxa associated with time elapsed since last treatment.

**Reply to reviewer:**

We thank the reviewer very much for noticing this wording that can indeed be misleadning. We have changed the section and hope the reviewer agrees it now reflect the results accurately. The end of the subsection now reads:

*At the genus level, a lower abundance of the genera Anaerostipes and Fusicatenibacter (Firmicutes), and a greater abundance of the genus Parasutterella (Proteobacteria), were associated (p.adj<0.05) with the time elapsed since last infusion in UC specifically, while only Parasutterella was associated across the full cohort (p.adj=1e-10).*

As we have added a sentence to the section we needed to also shorten the abstract in other places and thus other adjustments have been made aiming to keep the meaning of the abstract intact.

**Comments with replies for reviewer Jakub Hurych, M.D., Ph.D.**

Firstly we would like to thank the reviewer for this time and for the apology. While we indeed found the wording somewhat harsh, we do appreciate that written words can come across harsher than intented. No harm was done.

Row 79 (and also 392-409 and 480-495)

1.“(p.adj<0.1) “ is no statistical association, this is only below 0.05.

There is no doubt about an association between the genera and the time since the last infusion in UC ( p.adj=0.037, 0.037, and 0.037; line 391). But for CD, there is no mention about statistical significance between the three genera and time since treatment, as line 395 shows a p-value <0.1 – it is only nominal and not statistical; I would mention it in the results, but discuss and highlight only the statistically significant results.

Similarilly, in the discussion, all is discussed as a clear significance between the three genera and time since treatment in both UC and CD. But again, this is not supported by the results. I would like to see Table 1 with all the modelling results, but I was not given the opportunity to see it (I asked via email, but with no response), unfortunately. But if the adjusted p-value for the relation between Parasuterella, Fusicatenibacter and Anaerostipes in CD is not below 0.05, it is not statistically significant and should be discussed as that.

To sum up, I would kindly ask the authors for either an explanation to or a correction in this regard.

**Reply to reviewer:**

This same point was made by reviewer 1 for the abstract and has been addressed as outlined above. For the result section of these taxa around lines 380-397 we only do call the associations significant that have p.adj<0.05 (being those in UC) and do spend some lines highlighting that the association differed between the two subtypes. We do mention other associations but do not call them significant. In the discussion section we have indeed missed to update the wording and thank the reviewer for noticing. It has not been updated to read:

*We also found a lower abundance of Anaerostipes (Firmicutes) and Fusicatenibacter (Firmicutes), and a higher abundance of the Proteobacteria Parasuterella, was significantly correlated with the time since the last IFX infusion in UC (p.adj<0.05) while only the association for Parasuterella was significant in the full cohort (p.adj=1e-10).*

Row 97

It seems confusing: … “before diversity decreases again” - I got the point, but it took me a while; I would suggest the authors consider different wording to make the diversity dynamics statement clear enough.

**Reply to reviewer:**

We agree the wording was somewhat hard to follow. We have rephrased to now include the following:

*Our study illustrates that the composition of the microbiome is dynamic and alterable. Alpha diversity first increases rapidly (within the first four weeks) after treatment with infliximab, and then decreases again prior to the next infusion (at 8-12 weeks).*

Row 425-6

I appreciate the idea of summarising the main point of the article, but it seems a word might be missing or I did not understood the meaning.

**Reply to reviewer:**

We agree that sentence was disconnected, and we have changed it to the following and hope it now hold the intended point for the discussion of the pattern observed for alpha diversity:

*The observed diversity dynamics of this study likely reflect the concentration of IFX that will initially increase and then decrease over time after infusion, and consequently, the anti-inflammatory effect of the treatment, and thus the associated change in the microbiome, will depend on time since treatment.*
